# Rhythmic Aortic Contractions Induced by Electrical Stimulation *In Vivo* in the Rat

**DOI:** 10.1371/journal.pone.0130255

**Published:** 2015-07-01

**Authors:** Niaz Sahibzada, Allen W. Mangel, Jaclyn E. Tatge, Kenneth L. Dretchen, Michael R. Franz, Renu Virmani, Richard A. Gillis

**Affiliations:** 1 Department of Pharmacology & Physiology, Georgetown University School of Medicine, 3900 Reservoir Road, NW, Washington, DC, 20007, United States of America; 2 RTI Health Solutions, 3090 Cornwallis Drive, Research Triangle Park, NC, 27709, United States of America; 3 Cardiology Division, Veterans Affairs Medical Center, 50 Irving St, NW, Washington, DC, 20422, United States of America; 4 CVPath Institute, Gaithersburg, Maryland, 20878, United States of America; Cinvestav-IPN, MEXICO

## Abstract

For over a century, the behavior of the aorta and other large arteries has been described as passive elastic tubes in which no active contraction occurs in the smooth muscle wall. In response to pulsatile pressure changes, the vessels undergo a 'passive' elastic dilatation–contraction cycle, described as a “Windkessel” effect. However, Mangel and colleagues have presented evidence that is contrary to this view. They reported that in the rabbit, the aorta undergoes rhythmic 'active' (contraction) during the cardiac cycle; but these findings have been largely ignored. In the present study, we observed spontaneous contractions in synchrony with the heartbeat in another species (rat). In addition we demonstrate that aorta contractions are of neurogenic origin. Electrical stimulation of the aorta evoked contractions that occur at a rate that is in the range of the animal's heartbeat and are suppressed by tetrodotoxin and the alpha-adrenergic receptor blocker, phentolamine. Altogether, these findings indicate that aortic contractions are under neural control from the heart.

## Introduction

Smooth muscle and endothelial cells are active components of large vessels and contribute to their compliance [1]. However, one of the fundamental platforms underpinning the current understanding of the cardiovascular system is that the smooth muscle wall of the aorta and other large vessels behave as elastic tubes [2–7] with the smooth muscle component of the wall dilating and constricting in a passive manner. This is known, for the aorta and other large arteries, as the “Windkessel” effect that is considered an elastic dilatation-contraction cycle. However, a periodic rhythmicity in the smooth muscle wall of the aorta (pulse synchronized contractions [PSCs]) has also been shown to occur in synchrony with the cardiac cycle [8–11]. Whether PSCs result from a movement artifact has been thoroughly investigated and discounted [10]. The reasons for discounting are: (a) PSCs persist following bleeding of animals in which there were no pulsatile pressure changes [10]; (b) PSCs are abolished following removal of the right atrium even if cardiac contractility persists [10] and (c) electrical stimulation of the right atrium during periods when the heart is mechanically refractory yields an ectopic PSC without a corresponding ectopic pulse pressure wave [9]. In spite of this evidence, rhythmic 'active' contractile behavior of the aorta has been ignored. Here, using the rat as the experimental subject, we confirm earlier results obtained in other species [9,10] that the smooth muscle wall of the aorta is able to undergo rapid phasic contractions in synchrony with the cardiac cycle. We also show for the first time that direct electrical stimulation of the aorta in the *in vivo* rat model is capable of producing rapid contractions that are neurogenic in origin.

## Materials and Methods

### Animals

Experiments were performed on male Sprague-Dawley rats weighing 340–380 grams (Harlan Laboratories) in accordance with the National Institutes of Health guidelines for use of animals in research and with the approval of the Georgetown University Animal Care and Use Committee (Permit Number: 13–009).

### General Procedures

Animals were anesthetized with an intraperitoneal injection of urethane (1.5 g/kg) dissolved in 0.9% saline. Body temperature (37°C) was controlled with an infrared heat lamp. After a surgical depth of anesthesia was confirmed, rats were intubated and placed on a respirator. Next, a laparotomy was performed and extended into a bilateral anterolateral thoracotomy. The descending thoracic aorta was isolated and ligated at the level of the apex of the heart. A second ligature was placed at the distal end of the aorta (approximately 2 cm from the first).

### Experimental Preparation for Studies of Correlative Recording of Spontaneous Aortic Contractions and ECG

A Millar Catheter (3.0 French; AD Instruments, Inc) was used to record pressure in the thoracic aorta. It was inserted through a small cut into the vessel from the distal ligated end and tied in place after it was adjusted to rest approximately 1cm from the proximal ligature. While pressure changes in the aorta was one method of recording spontaneous contractions, in some experiments, they were recorded using an isometric force transducer (MLT0202, AD Instruments) connected by a 4.0 silk thread attached to the aorta. Blood pressure was also monitored by cannulation of the carotid artery, which was done to keep us informed about the physiological condition of our experimental preparation during the course of the experiment, and to confirm that a subset of rats were successfully bled. To monitor the ECG, differential recordings were made using two needle electrodes placed subcutaneously, one in the right forelimb and the other placed in the left hindlimb (low pass filter setting at 100 Hz).

### Experimental Preparation for Studies of Electrical Stimulation and Recording of Spontaneous and Evoked Aortic Contractions

**Fig 1:**
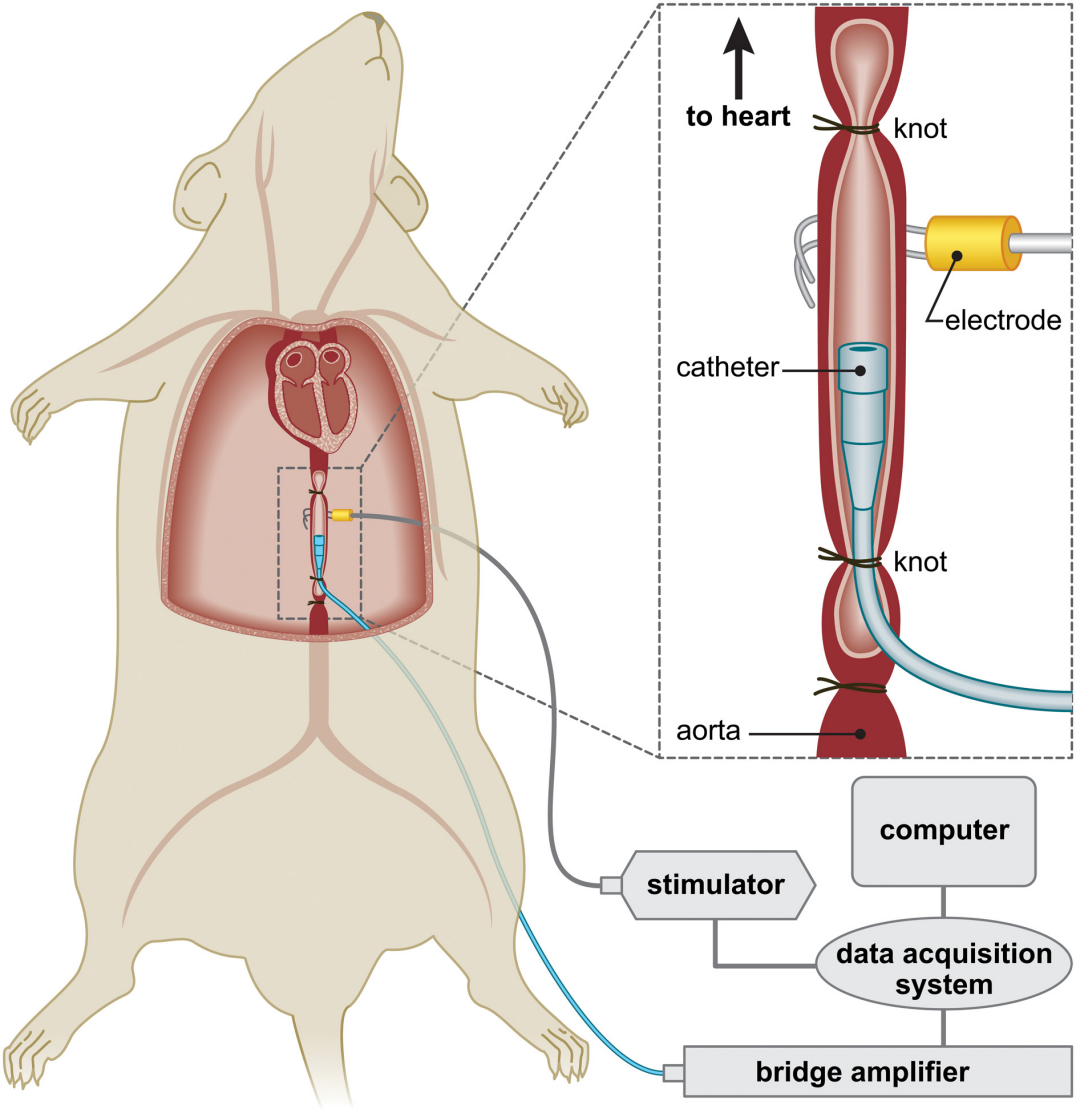
Schematic illustration of the set-up performing electrical stimulation of the aorta. To elicit aortic contractions, the aorta was directly stimulated with a 5s pulse train at different frequencies (pulse width, 2ms) at a 4 min inter-stimulus train using a constant voltage generator. Once two stable series of contractions were obtained at the same frequency, the aorta was exposed to different drugs by direct application into the thoracic cavity in 1ml increments. That is, an additional 1 ml of drug solution was added to the thoracic cavity. The volume of mineral oil and/or Tyrode's solution placed in the cavity was approximately 5–7 ml. This was followed by a series of stimulations over 5 s that were spread 4 min apart.

Proximal to the second ligature placed at the distal end of the aorta (approximately 2cm from the heart), a small incision was made in the aorta, which was cannulated with a 16 gauge gavage needle attached to a silicone tubing. After cannulation, a hooked bipolar electrode was positioned underneath the aorta proximal to the gavage needle. The aorta was then inflated with warm Tyrode’s solution to produce a baseline pressure of 0.8–1.6 kPa (6–12 mmHg), measured by a pressure transducer (sensitivity 5μV/V/mmHg; low pass filter set at 100Hz), which was coupled to a computerized data acquisition system (PowerLab AD Instruments, Colorado Springs, Co.). The thoracic cavity was then filled with warm (approximately 37°C) mineral oil or Tyrode's solution (pH 7.4; 300 mOsm) with the following composition (in mM): 121 NaCl, 2.5 KCl, 26 NaHCO_3_, 1.25 NaH_2_PO_4_, 2 CaCl_2_, 1 MgCl_2_, 5 HEPES and 10 glucose. The ECG was recorded as described above. Fig 1 illustrates the rat preparation used for recording aortic contractions.

**Fig 2:**
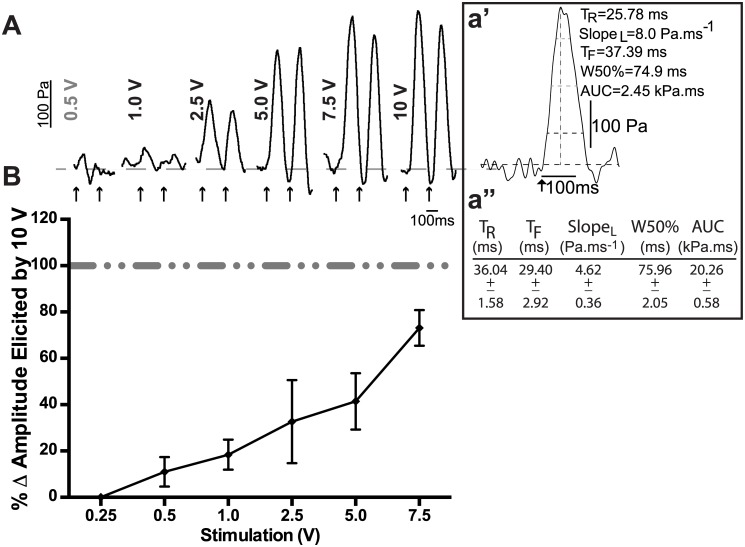
Effect of varying stimulation voltage on magnitude of the contractile response of the aorta. Voltage was varied from 0.25–7.5V, whereas frequency and pulse duration were kept constant at 5Hz and 2ms, respectively. (A) Representative tracing of electrically induced contractions of the aorta in response to varying voltage stimuli (two/voltage; bars) and peak parameters (a') of a 10V induced single contraction and (a") the mean ± SEM of 5 contractions per aorta in three animals (arrow; TR = rise time, TF = fall time, SlopeL = leading slope, W50% = half-width, AUC = area under the curve). (B) Graph showing the stimulus-response profile of aortic contractions in relationship to the percent change in amplitude elicited by 10V (n = 4).

To consistently induce single contractions of the aorta, we employed frequencies of 1.7 Hz (to approximate a heart rate of 100 beats/min) and 5 Hz (to approximate a heart rate of 300 beats/min). These stimulation rates did not change the basal tone of the aorta. Fig 2 illustrates a stimulation-response curve that was derived using a 5 Hz frequency with varying voltages (0.25V-10V). The typical profile of electrically-evoked neurally mediated contraction had a relatively fast rise-time and recovery compared to that reported for direct muscle-stimulated vascular contraction [12]. For our studies, to assess the effect of drugs on stimulation-induced contractions, we utilized the 10V stimulation strength because it elicited repeatable aortic contractions of similar amplitudes. Fig 2: Effect of varying stimulation voltage on magnitude of the contractile response of the aorta.

### Data Analysis

Assessment of evoked aortic phasic contractions was determined in conjunction with electrical stimulation pulses. A continuous 6s time frame was the “experimental window” within which response to electrical stimulation was evaluated. All data were analyzed using a paired t-test or one-way repeated measures ANOVA to assess significant differences between control and treatment conditions. Treatment interactions were assessed by the Bonferroni’s multiple comparisons test. All data are expressed as means ± SEM.

### Drugs

The drugs used were purchased from the following companies: urethane and phentolamine from Sigma-Aldrich, and tetrodotoxin (TTX) from Ascent Scientific. All drugs were constituted in warm Tyrode's solution.

## Results

**Fig 3:**
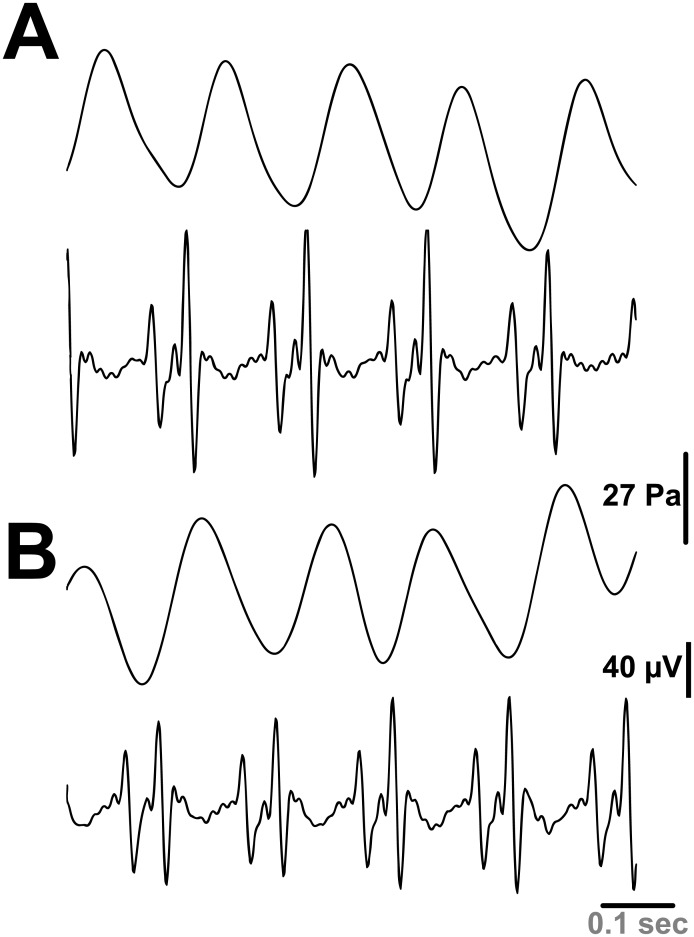
Spontaneous rhythmic contractions (pulse pressure) of the aorta correlated to ECG. (A, B) Representative tracings of aortic contractions (upper panel) and ECG recording (lower panel) before (A) and after (B) bleeding the rat.

Our goal in the first series of experiments was to confirm the earlier findings of Mangel and colleagues [10] obtained in the rabbit, namely, that the rat aorta undergoes spontaneous rhythmic contractions in synchrony with the heartbeat. That this was the case is shown in Fig 3. The traces displayed in part A of the figure are aortic contractions (upper) and the ECG signal (lower). These recordings were made with a Millar catheter placed in the aorta and obtained prior to bleeding the animal. Note the correlation of aortic contraction with the ECG (i.e., the heartbeat). Part B of the figure shows these traces after bleeding the animal. In this condition, rhythmic contraction of the aorta would not be due to transference of contractile activity from other parts of the vascular system. Yet, rhythmic contractions do occur and are correlated with the heartbeat. While these are data from a single rat, the same result was obtained in 3 of 4 rats where PSCs persisted when the rats were bled, and pulsatile pressure changes in the vascular system were eliminated. Removal of the right atrial appendage eliminated PSCs in 2 of 3 rats studied while cardiac contractions persisted.

**Fig 4:**
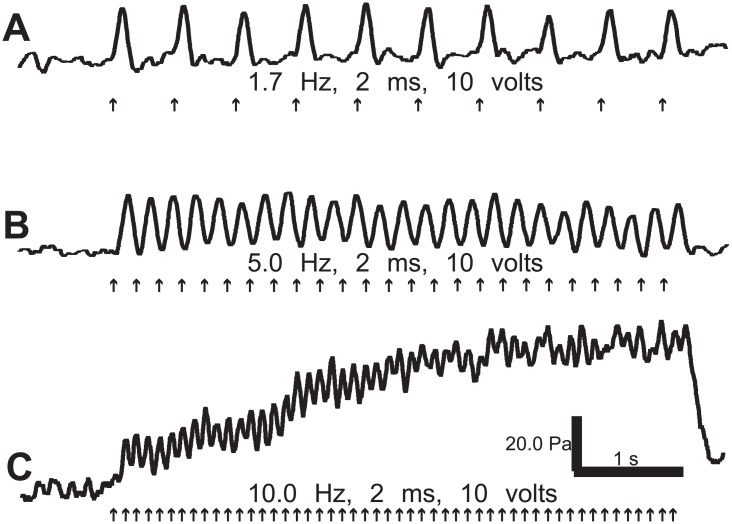
Contractile activity of the *in vivo* aorta following different frequencies of electrical stimulation. Stimulation was applied for 2 ms duration, 10V intensity over a 5s pulse train. (A-C) Representative recordings of phasic contractions in response to 1.7 Hz (A), 5 Hz (B) and 10 Hz (C) stimulation frequencies. [Note: the fast contractions superimposed on a tonic rise in pressure in C.]

The primary goal of the present study was to assess whether the smooth muscle wall of the aorta would undergo rapid contractions upon electrical stimulation at frequencies between 1.7 and 5 Hz. Employing this procedure (see Fig 1), we observed rhythmic phasic contractions (n = 17; Fig 4A and 4B) with the magnitude of the electrically induced contractions larger than the spontaneous occurring PSCs. Further increases in frequency (i.e., to 10 Hz) led to tonic tension changes in the aorta (Fig 4C, n = 3).

**Fig 5:**
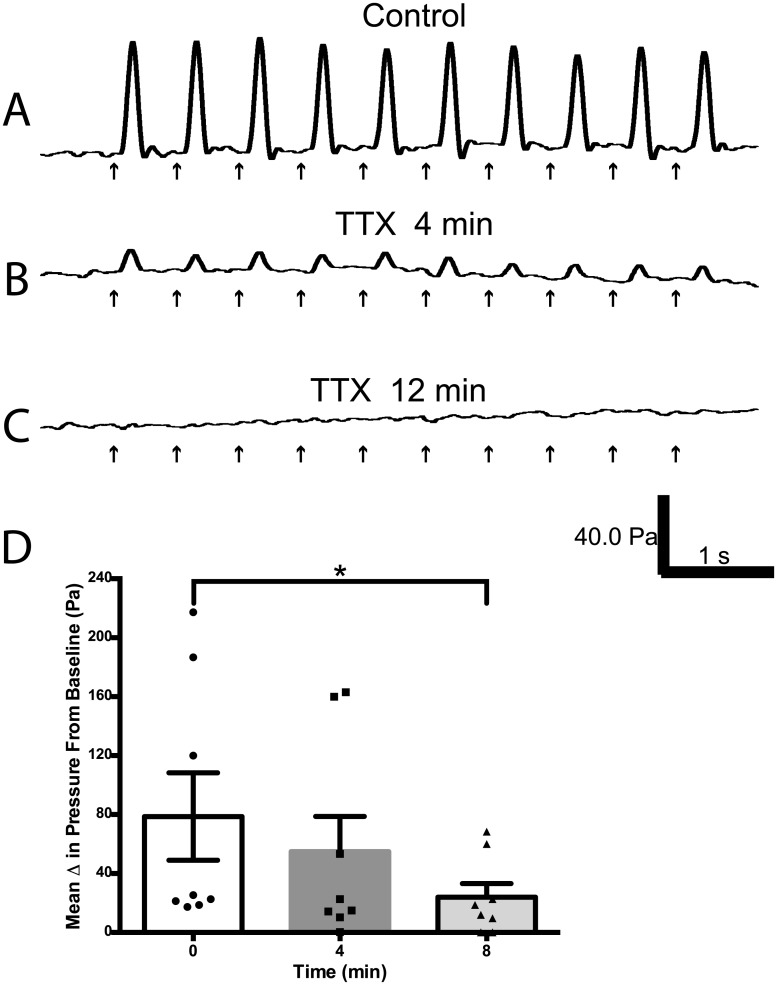
Tetrodotoxin (TTX) inhibits electrically evoked aortic contractions (n = 8). (A) Rhythmic phasic contractions electrically induced by a 5s pulse train (1.7 Hz, 2 msec, 10 volts). Topical application (1 mL of 2 μM) of TTX reduced contraction amplitude after a 4-minute exposure (B), and, with continued exposure, contractions were eliminated 12 minutes later (C). (D) Graph showing progressive inhibition of electrically induced contractions of the aorta after initial application of TTX. * Significance p<0.05. [Note: The 12 min time point was not used in the analysis as there was complete inhibition of the electrically-induced contraction of the aorta in all the animals.]

Once obtaining these electrically induced contractions of the aorta, the next question was whether they were the result of electrical stimulation of the nerves innervating the aorta or were they due to direct stimulation of its smooth muscle? To determine this, we assessed whether topical application of TTX would affect electrically induced contractions. Illustrated in Fig 5B and 5C are aortic contractions evoked by electrical stimulation recorded at 4 and 12 min intervals after topical application of 1ml of 2μM TTX. As can be noted, TTX produced a complete block of the electrically induced contractions. Data obtained with TTX are summarized in Fig 5D, and indicate a significant block of electrically induced contractions of the aorta at 8 and 12 minutes after application of TTX.

**Fig 6:**
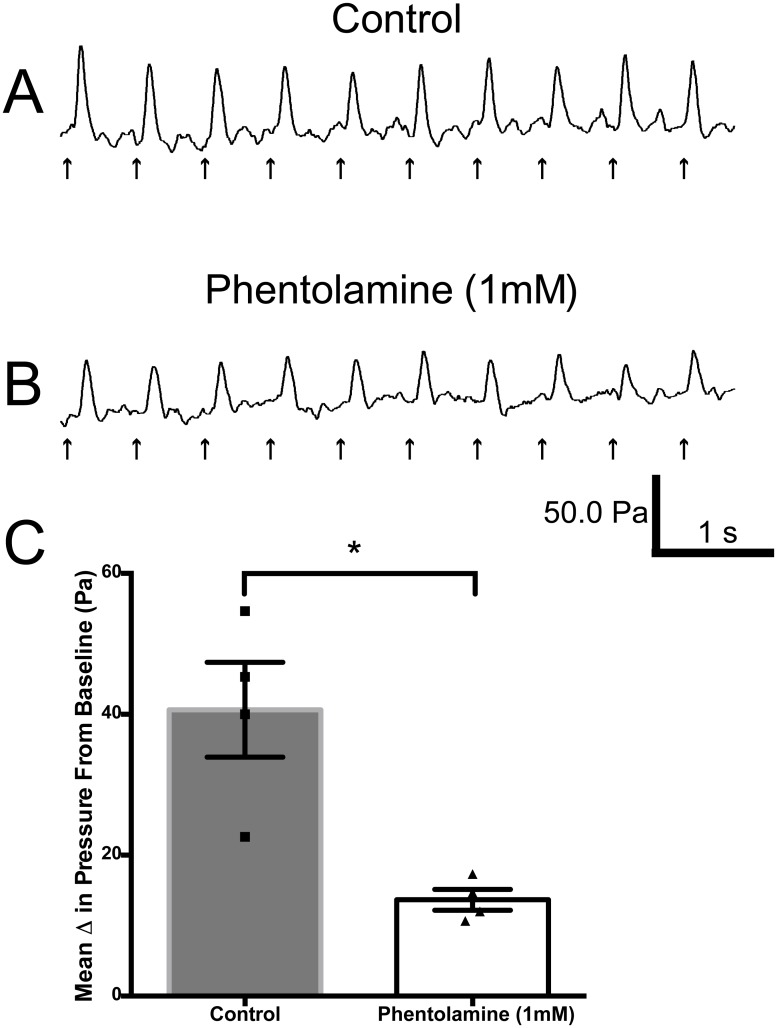
Suppression of electrically evoked aortic contractions by phentolamine. (A) Rhythmic phasic contractions elicited in the aorta by a 5s pulse train (1.7 Hz, 2 msec, 10 volts), the amplitude of which was reduced by topical application of 1 mL of 1 mM phentolamine (B). (C) Graph illustrating the mean change in pressure from baseline induced by electrical stimulation of the aorta in the absence and presence of phentolamine (n = 4). * Significance p<0.05.

To determine the nature of the neurally induced aorta contraction evoked by electrical stimulation, we evaluated whether topical application of the non-specific α-adrenergic receptor blocker, phentolamine would prevent the response. Following electrically evoked control aortic contractions; 1ml of 1.0 mM phentolamine was topically applied to the aorta. As can be noted in Fig 6, phentolamine application in all but one rat significantly reduced or eliminated the electrically induced aortic contractions. Additionally, when the phentolamine application reduced the response but failed to abolish it, subsequent application of the TTX solution inhibited the remaining electrically induced contraction of the aorta.

## Discussion

It has long been understood that the smooth muscle wall of large arteries is not capable of undergoing rhythmic activation during the cardiac cycle. In the current series of experiments, we demonstrate for the first time that fast rhythmic electrical stimulation of the aorta results in induction of fast rhythmic contractions. The phasic contractions could be entrained by stimuli occurring at a rate of up to 300 per minute (i.e., 5Hz stimulation), which is well within the range of rat spontaneous heart rate. These observations confirm that the smooth muscle component of large conduit arteries is able to undergo rapid phasic contractions, an important contrast to the Windkessel description of the vessels' behavior in response to changing blood pressure. Furthermore, sensitivity of the contractions to TTX establishes a neurogenic, as opposed to a myogenic basis for the events. Studies with phentolamine show that the excitatory pathway is at least in part mediated by norepinephrine released from sympathetic nerve terminals.

Others have demonstrated that the smooth muscle wall of the large arteries may generate rhythmic contractions *in vitro* either spontaneously or following potassium depolarization [13,14]. These events, however, occur at a significantly slower rate than the cardiac cycle. When present, the contractions are often sensitive to application of calcium channel blockers and are associated with slow rhythmic changes in membrane potential; therefore, they represent myogenically driven events. What we have established by our study is that electrical stimulation of the aorta results in induction of fast rhythmic contractions, which is neurogenic in origin. Our evidence for the neural basis of the contractions rests on our use of TTX and phentolamine. The concentrations of both drugs seem high and may be exerting a non-specific membrane action. We do not believe this to be the case. Our selection of a 2μM concentration of TTX was based on several considerations. First, dilution of the toxin applied topically to the smooth muscle vasculature (SMV) of the aorta was expected to achieve nanomolar concentrations due to its addition to the accumulated mineral oil or Tyrode's solution in the thoracic cavity (approximately 5 to 7ml that was used to constantly irrigate the tissue and prevent it from drying). Second, blockade of sodium channels in the SMV at the diluted concentrations was not thought to significantly contribute to TTX inhibition of the aorta contractions in our study as 1 to 6μM fails to suppress induced or spontaneous rhythmicity in other SMV [15,16]. The rationale for the dose of phentolamine was similar to that of TTX and in our *in vivo* preparation reached a likely dilution of approximately 200μM. [Note: in all cases, 2μM TTX inhibited the remaining electrically induced contractions of the aorta that could not be blocked by phentolamine.] As to the remaining electrically induced contractions of the aorta that remained after phentolamine, these may have been due to activation of purinergic receptors. It has been reported that ATP and norepinephrine are co-stored and co-released from sympathetic nerves, and that ATP can cause vasoconstriction [17].

Evidence for norepinephrine mediating the electrical stimulation–induced aortic contractions runs counter to the finding of Patil, et al. [18] who report the absence of adrenergic nerves in the rat thoracic aorta. However, in a later study by Nilsson et al. [19], nerve fibers were reported to innervate the abdominal aorta of the rat. Furthermore, electrical stimulation of conduit vessels elicited contractions, which were described of neurogenic origin. Our studies with electrical stimulation focused on the thoracic aorta of the rat and data obtained are consistent with those reported for the abdominal aorta by Nilsson et al. [19].

## Conclusions

Our results confirm the presence of aortic PSCs and demonstrate for the first time that direct stimulation of the aorta can trigger rhythmic phasic contractile activity that is neurogenic in origin. Although the precise role of PSCs has not yet been elaborated, the phasing of the contraction wave with the pulse wave may suggest that PSCs tend to limit the distension of the vessel wall [2]. Limiting this distension may serve to reduce the Laplacian forces acting on the wall to protect against dissection and aneurysm formation. This precise and important function needs to be studied.
